# Frontal Alpha Asymmetry and Theta Oscillations Associated With Information Sharing Intention

**DOI:** 10.3389/fnbeh.2018.00166

**Published:** 2018-08-02

**Authors:** Nastassja L. Fischer, Rafael Peres, Mario Fiorani

**Affiliations:** ^1^Laboratory of Cognition Physiology, Institute of Biophysics Carlos Chagas Filho, Federal University of Rio de Janeiro, Rio de Janeiro, Brazil; ^2^Department of Morphological Sciences, Medical School Souza Marques, Rio de Janeiro, Brazil

**Keywords:** social media, social networking, stimuli propagation, attention, social conformity, decision-making, EEG

## Abstract

Social media has gained increasing importance in many aspects of everyday life, from building relationships to establishing collaborative networks between individuals worldwide. Sharing behavior is an essential part of maintaining these dynamic networks. However, the precise neural factors that could be related to sharing behavior in online communities remain unclear. In this study, we recorded electroencephalographic (EEG) oscillations of human subjects while they were watching short videos. The subjects were later asked to evaluate the videos based on how much they liked them and whether they would share them. We found that, at the population level, subjects watching videos that would not be shared had higher power spectral density (PSD) amplitudes in the theta band (4–8 Hz), primarily over the frontal and parietal sites of the right hemisphere, than subjects watching videos that would be shared. Previous studies have associated task disengagement with an increase in scalp-wide theta activation, which can be interpreted as a mind-wandering effect. This might suggest that the decision to not share the video may lead to a more automatic/effortless neural pattern. We also found that watching videos that would be shared was associated with lower PSD amplitudes in the alpha band (8–12 Hz) over the central and right frontal sites, and with more negative scores of frontal alpha asymmetry (FAA) index scores. These results may be related to previous work linking right-sided frontal EEG asymmetry to the pursuit of social conformity and avoidance of negative outcomes, such as social isolation. Finally, using support vector machine (SVM) algorithms, we show that these EEG parameters and preference rating scores can be used to improve the predictability of sharing information behavior. The information sharing-related EEG pattern described here could therefore improve our understanding of the neural markers associated with sharing behavior and contribute to studies about stimuli propagation.

## Introduction

Being a member of a social group has been recognized as a crucial factor for human survival throughout the ages (Baumeister and Leary, 1995; Lieberman and Eisenberger, 2009) since it confers an evolutionary advantage by facilitating access to food and ensuring safety (Adolphs, 2009). People who have good social interaction skills are likely to have better opportunities and to adapt faster to an ever-changing environment. Social interaction involves sharing information with others, and is generally acknowledged as a core contemporary mechanism for innovation and human development (Scholz and Falk, in press). The emergence of online social networks over the past decade has boosted such social interactions. Social media networks can be defined as “a group of internet-based applications that build on the ideological and technological foundations of Web 2.0, and that allow the creation and exchange of user-generated content” (Kaplan and Haenlein, 2010:61). In a recent survey conducted by the Pew Research Center (2017), 76% of adult internet users from 40 countries stated that they used at least one type of social network. Content shared on social media platforms may influence human interaction on an individual and a broader societal level (Van Dijck, 2013). However, little is known about which personal variables, particularly neurophysiological factors, are involved in information sharing behavior. Most studies in this area rely on self-reported results and/or on content characteristics (Berger and Milkman, 2012; Stieglitz and Dang-Xuan, 2013; Ferrara and Yang, 2015). However, this type of approach does not account for implicit (unconscious) cognitive processes that could be involved in the sharing decision. Thus, there is a need for neuroscientific studies to uncover the fundamental mechanisms underlying information sharing.

Studies of the brain mechanisms that influence how ideas and information are propagated in online and offline environments are in their infancy (as reviewed by Falk and Bassett, 2017). Nevertheless, some empirical evidence from functional magnetic resonance imaging (fMRI) studies suggests that information sharing behavior is related to three primary domains: inferring the thoughts and desires of others (i.e., mentalizing), self-referential cognition and social reward processing (Meshi et al., 2015) and likely involves brain areas such as the temporoparietal junction (TPJ) and dorsomedial prefrontal cortex (dmPFC) in those processes (Falk et al., 2013; O’Donnell et al., 2015; Baek et al., 2017; Scholz et al., 2017). However, although fMRI has excellent millimeter-range spatial resolution and provides a good estimate of the location of neural processes, it has some disadvantages. For example, some authors already arose the issues about the role of fMRI hemodynamic signal as an indirect indicator of neural activity (Logothetis, 2008; Hari and Kujala, 2009) and the need of a sophisticated infrastructure to run fMRI experiments (Glover, 2011). The drawbacks of fMRI in combination with the relative paucity of neuroscientific studies about information sharing behavior using non-invasive neuroimaging techniques prompted us to investigate the use of electroencephalography (EEG) as an alternative method of correlating brain activity with sharing behavior.

EEG directly records brain activity at the millisecond (ms) level and is thus generally acknowledged to provide a direct measure of the dynamic interaction between different brain systems as they are occurring (Ward, 2003; Buzsáki and Draguhn, 2004; Lopes da Silva, 2013; Cohen, 2014). Additional advantages of EEG include its flexibility in accommodating a range of experimental designs and subjects (e.g., healthy subjects, patients and children); its portability, which permits analysis outside a laboratory setting; and its relatively low cost (Loo et al., 2015). EEG currents can be quantified and classified into five bands of differing frequency (also known as EEG rhythms): delta (1–4 Hz), theta (4–8 Hz), alpha (8–13 Hz), beta (14–30 Hz) and gamma (>30 and typically <100 Hz; Pizzagalli, 2007; Urigüen and Garcia-Zapirain, 2015). However, the precise range assigned to these bands can vary across studies (Klimesch, 1999; Wang et al., 2015). The standard method of quantifying frequency band oscillations is the power spectrum analysis or power spectral density (PSD), which describes the distribution of signal power at differing frequencies, or the “frequency content” of the signal (Dressler et al., 2004).

Substantial evidence supports the use of EEG to investigate how frequency band types are associated with particular cognitive processes. For example, studies have shown that theta and gamma rhythms are associated with memory processes such as retrieval and encoding (Klimesch, 1999; Nyhus and Curran, 2010), alpha and gamma rhythms are related to visual processing prioritization (Jensen et al., 2014), and whole-scalp gamma frequency synchronization is associated with consciousness (Doesburg et al., 2008). EEG parameters can also be useful for analyzing preference selection and predicting behavior. Vecchiato et al. (2011) found that theta-band activity in the right hemisphere was higher when participants watched videos they did not like compared with those they did like. Wang et al. (2016) showed that video commercials containing a structured narrative significantly affected the viewer’s product branding preference compared with other commercials and induced higher theta power in the left frontal and bilateral occipital regions and higher gamma power in the limbic system. Christoforou et al. (2017) demonstrated that analysis of beta and gamma EEG bands could successfully predict box-office performance of newly released movies. Finally, Park et al. (2018) analyzed EEGs to assess participants’ abilities to predict movie poster preferences of another participant about whom they had minimal information, and they discovered a time-frequency-related relationship between beta-band frequency at right temporal sites and prediction accuracy. Taken together, these studies support the utility of EEG recordings to study neural correlates of a range of behavioral outcomes.

Another widely used method in cognitive neuroscience studies of behavior is the use of indexes that combine two or more EEG variables (Vecchiato et al., 2011; Yener and Başar, 2013; Cheron et al., 2016). One example is frontal alpha asymmetry (FAA), which is defined as the difference between right and left alpha activity over frontal regions of the brain (Davidson et al., 1990) and is thought to be a measure of the propensity to adopt approaching vs. withdrawing behavior (Coan and Allen, 2004). More specifically, greater left frontal activity (lower alpha power) is associated with an increased tendency to approach or more intensely respond to affectively positive stimuli, whereas greater right frontal activity (or, possibly, lower left frontal brain activity) is associated with an increased tendency to withdraw or more intensely respond to affective negative stimuli. Thus, alterations in FAA detected by EEG can be correlated with emotional/motivational personality traits (Reznik and Allen, 2018). FAA has also been correlated with various aspects of social decision-making and empathy, pointing to a possible link between right hemisphere lateralization and social-oriented behaviors (Hecht, 2014). For example, Sabbagh and Flynn (2006) showed that mental state decoding skills (i.e., judgments about others’ psychological states) were stronger in those with right-sided compared with left-sided frontal alpha activation. Furthermore, the degree of relative right mid-frontal activation predicted performance on a mental decoding task. In another study, children who exhibited right-sided frontopolar EEG activity during an emotion-eliciting task showed empathic concern when they observed pain being expressed by the experimenter (Light et al., 2009). Similarly, Tullett et al. (2012) found that baseline measures of right frontal asymmetry were a significant predictor of empathic concern, as indicated by feelings of sadness. These authors suggested that higher right FAA might increase sensitivity towards others’ suffering. Since sharing behavior may involve inferring the thoughts and desires of others (Scholz and Falk, in press) and can also be related to motivation (approach vs. avoidance behavior), it is possible that FAA could also be associated with information sharing behavior.

Correlating EEG oscillations and indexes with specific cognitive, affective and decision-making tasks may allow us to better identify the types of information being processed with temporal precision. This information could be applied to brain–computer interface methods, which allow typically covert aspects of the mental state to be assessed with little-to-no interference on the task at hand (van Erp et al., 2015). EEG analysis could also be used to improve prediction of behavioral outcomes, since pre-existing judgments and socially presumed expectations have less influence on EEG oscillations than on traditional self-reported measures such as questionnaires (Telpaz et al., 2015).

Although information sharing is increasingly prevalent in the context of social media networks, little is known about its neural correlates. To address this, we explored brain parameters related to information sharing behavior by recording EEGs when subjects were watching short videos that they subsequently decided they would or would not share with others. This video-based approach has been used increasingly in neuroscience research in recent years (Ki et al., 2016). We then identified which EEG features differed when watching videos that would vs. would not be shared and applied a support vector machine (SVM) algorithm to identify the precise features could accurately predict information sharing behavior. Our goal was to improve our understanding of neural correlates of social-oriented behavioral patterns such as information sharing.

## Materials and Methods

### Subjects

The study consisted of two experiments. First we performed a pre-selection experiment in which a small cohort (*n* = 10, “Stimuli Selection” section) of subjects watched a large number of videos and then answered questions. Second based on their responses, the video pool was narrowed to 100 for viewing in the main experiment with a larger cohort (*n* = 23, “Stimuli Presentation and Experimental Design” section). The pre-selection cohort consisted of 5 men and 5 women aged 21–32 years (men 24.8 ± 3.7 years, women 24.1 ± 2.6 years, mean ± standard deviation [SD]). The cohort for the main experiment consisted of 23 additional subjects (12 men/11 women) aged 18–31 years (men 23.2 ± 3.50 years, women 23.1 ± 3.45 years).

All participants had a normal or corrected-to-normal vision; were not left-handed (assessed by the Edinburgh Handedness Inventory; Oldfield, 1971); and were free of diagnosed psychiatric disorders (assessed by the personal data formulary). We also only included participants with a Beck Depression Inventory score >18 to exclude subjects at risk for depression. Participants were unpaid volunteer undergraduate and graduate students at the Federal University of Rio de Janeiro. This study was carried out in accordance with the recommendations of the Comissão Nacional de Ética em Pesquisa (National Research Ethics Commission—CONEP). The protocol was approved by the Comitê de Ética em Pesquisa do Hospital Universitário Clementino Fraga Filho e da Faculdade de Medicina da Universidade Federal do Rio de Janeiro (Ethics Institutional Review Board of the University Hospital Clementino Fraga Filho and of the Medicine School of Federal University of Rio de Janeiro; protocol code: CAAE 02761212.0.0000.5257). All subjects gave written informed consent in accordance with the Declaration of Helsinki.

### Stimuli Selection

The 10 participants each watched 252 short video clips one time. The videos were downloaded from YouTube.com and Vimeo.com and edited to a maximum length of 10 s. After watching each video, the participants were asked: (1) if they liked the video (“yes” or “no”); (2) how much they liked the video (1–5 Likert scale of liked/disliked); and (3) if they had seen the video before (“yes” or “no”). The videos were presented using Psychtoolbox Brainard (1997) for MATLAB (The MathWorks, Inc., Natick, MA, USA) in a soundproof room.

To select the final 100 videos to be used in the main experiment, we eliminated 25 videos that had previously been viewed by at least one subject. The remaining 227 videos were then ranked according to the frequency of “yes” answers to question (1) above. For the final selection, 25 videos were liked by 7–10 subjects, 50 were liked by 4–6 subjects and 25 were liked by 0–3 subjects. The final selection therefore included videos with a range of viewer preferences. Question (2) responses were not incorporated into the selection process because they reduced the final number of candidate videos to fewer than 100.

### Stimuli Presentation and Experimental Design

The main experiment was performed as described for the pre-selection experiment with some modifications. Participants were monitored throughout using a video camera to ensure compliance with the experimental design. To minimize the possibility that the subjects had previously viewed the videos, we confirmed that the 100 videos had a low view count at the host websites. The number of visualizations varied between 1–553,260 views. In comparison, the most watched video in the year this study was performed had 301 million views^1^.

**Figure 1:**
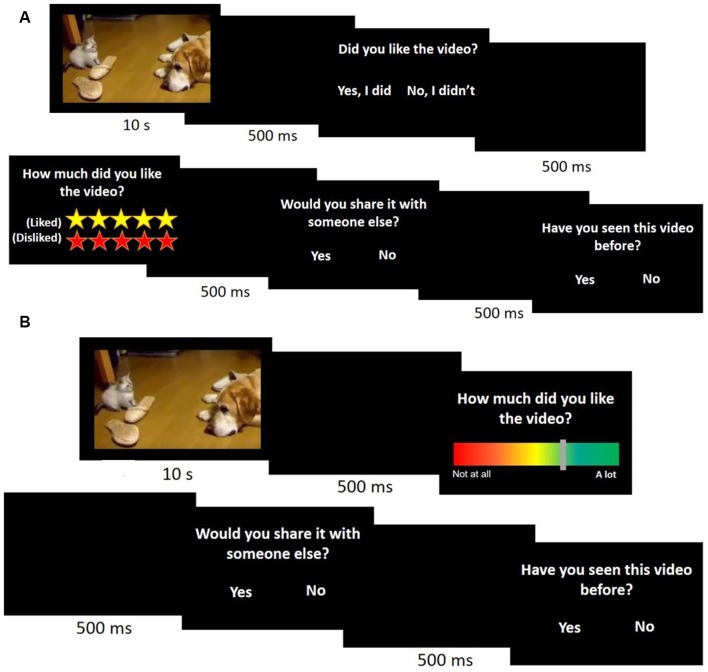
Main experimental design. A cohort of 23 participants watched 100 10-s videos and answered four (**A**, *n* = 11) or three (**B**, *n* = 12) questions immediately after viewing each one.

The main experiment consisted of two experimental designs, although the data were combined for analysis. The first cohort (*n* = 11; Figure 1A) viewed the videos and were then asked: (1) if they liked the video (“yes” or “no”); (2) how much they liked the video (Likert scale of liked/disliked, in which the number of yellow stars indicated how much participants liked the video and the number of red stars stated how much subjects disliked the video); (3) if they would share the video with another (“yes” or “no”); and (4) if they had seen the video before (“yes” or “no”). The second cohort (*n* = 12; Figure 1B) were shown the same videos but were asked only questions (2) through (4) in an effort to reduce the overall time taken for the task. In addition, the answer to question (2) (how much they liked the video) was changed from the Likert rating system to a color scale where red (score 0) indicated “not at all” and green (score 1) indicated “a lot” (Figure 1). The subjects were free to take as much time as necessary to answer the questions. The experimental design is shown in Figure 1.

### Behavioral Analysis

A non-parametric *t*-test (Mann-Whitney *U* test, two-tailed) was used to compare the “like” ratings of shared (S) vs. not shared (N-S) videos and the response times to answer the question “would you share the video?” We excluded response time outliers (greater than or equal to ±3 SD from the mean [*n* = 100]) and response times of <200 ms to avoid possible anticipation errors. We also recorded the sharing behavior of each participant based on the number of videos they decided to share. The behavioral analysis was performed using STATISTICA data analysis software, version 12 (StatSoft, Inc., Tulsa, OK, USA, 2014). *p* ≤ 0.05 was considered statistically significant.

### EEG Data Acquisition and Pre-processing

EEGs were recorded using a BNT36 EEG model (EMSA^®^, Rio de Janeiro, RJ, Brazil) at the following positions of the 10–20 system: Fp1, Fp2, F7, F3, Fz, F4, F8, T7, C3, Cz, C4, T8, P7, P3, Pz, P4, P8, O1, Oz, O2, A2 and A1. 10-20 electrode paste (D.O. Weaver and Co., Aurora, CO, USA) was applied to each electrode before placing it on the scalp. Electrode impedance was ≤5 kOhms. EEG signals were acquired at a 600 Hz sampling rate.

Data were pre-processed using EEGLAB toolbox (Delorme and Makeig, 2004) for MATLAB. Continuous EEG data were filtered using a 0.1–45 Hz band-pass filter and re-referenced using the grand average reference algorithm as implemented in the EEGLAB routine. EEG epochs were extracted using a time window of 10.7 s (0.5 s pre-stimulus to 10.2 s post-stimulus) and were divided according to the start of each video. Noisy trials were removed by visual inspection. EEG data were decomposed into independent components (ICs) using the runica function implemented in EEGLAB. Components representing eye movements and/or muscle contractions were excluded by visual inspection to remove artifacts from the neural data.

### EEG Data Analysis

EEG data analysis was performed using the MATLAB toolbox Fieldtrip (Oostenveld et al., 2011) and in-house MATLAB scripts. The PSD at each electrode was calculated in 1 Hz steps between 2 Hz and 30 Hz with a 7-cycle length sliding window and a single Hanning taper in 50 ms steps. This gave a decreasing time-window length as the frequency increased (e.g., 700 ms for 10 Hz and 350 ms for 20 Hz). For each channel, PSD values obtained during viewing of S and N-S videos were averaged for each EEG frequency band: (1) theta (4–8 Hz); (2) alpha (8–12 Hz); and (3) beta (12–20 Hz).

PSD values were log-transformed for group-level analysis. A cluster-based permutation test with a Monte Carlo approach (Maris and Oostenveld, 2007) was used to test for significant differences in PSD between the S (*n* = 316) and N-S (*n* = 1099) trials. Cluster-based statistics involves grouping of neighboring variables (here, *t*-values) into clusters and deriving characteristic values. In practice, values are only considered for inclusion in a cluster if they exceed a certain threshold (e.g., *p* value of <0.05 in univariate analysis). It is then possible to compute the clusters’ attributes and their associated probabilities (Pernet et al., 2015). Therefore, a *t*-statistic was calculated for each electrode for theta, alpha and beta frequency bands, with time averaged from 0 ms to 9000 ms during viewing of S and N-S videos. The final 1000 ms was not employed to eliminate possible artifacts caused by the anticipation of answering questions. Channel-frequency points with a *t*-value >95th percentile were retained. Adjacent frequency-channel points were clustered, and cluster statistics were calculated as the maximum sum of each cluster. A permutation test was then conducted, consisting of randomizing the trials for each condition (N or N-S) and recalculating a *t*-statistic. This procedure was repeated 2000 times. Finally, we obtained a reference distribution of cluster-based *t*-statistics and tested the distribution against the observed *t*-statistic. We also conducted a time-frequency analysis using cluster-based statistics as described before. Channel-frequency-time points with a *t*-value >95th percentile were retained.

For calculation of the FAA index, the normalized PSD of the alpha frequency band (8–12 Hz) from the left mid-frontal electrode F3 was subtracted from the right mid-frontal electrode F4 (lnPSD F4 − lnPSD F3). Because alpha power is inversely related to cortical activity (Gollan et al., 2014), a positive FAA index reflects greater left-sided frontal activity (lnPSD F3 > lnPSD F4); whereas negative values indicate greater right-sided activity (lnPSD F3 < lnPSD F4). The FAA index for S and N-S videos was compared using a permutation test.

### Classification of EEG Data

To determine whether the EEG data can be used to classify sharing intention, we used a SVM with a linear kernel (*C* = 1) from a libSVM library (Chang and Lin, 2013). The utility of SVMs for classifying brain states has previously been demonstrated (Mourão-Miranda et al., 2005). Time-averaged power of each frequency band with a significant difference between S and N-S videos and FAA indexes were used as features. Classification was performed using all 1415 trials as samples. To account for the imbalance between the number of S (*n* = 316) and N-S (*n* = 1099) trials, oversampling was performed with the S condition using the synthetic minority over-sampling technique (SMOTE; Chawla et al., 2002). This method consists of creating synthetic data points by taking the vector between one of the k nearest neighbors in feature space (we worked with five nearest neighbors), multiplying it by a random number between 0 and 1 and adding this point to the sample to create a new one.

To determine whether the EEG data plus self-reported measures are helpful in assessing sharing intention, we compared SVM performed with a self-reported measure (like/dislike scores) as the sole feature with SVM performed with the self-reported measure plus EEG data (theta- and alpha-band PSDs and FAA index) as features. All performance measures were obtained in a 10-fold cross-validation, and feature sets were divided into 10 mutually disjointed training (final number = 1978) and test (final number = 220) sets. The results are reported as accuracy, the sum of true positive (TP) plus true negative (TN) cases divided by the number of sample instances; and F1-score, which is a weighted average of the precision and recall and is defined as *F*1 = 2*(precision * recall)/(*precision + recall*), where *Precision* = *TP*/(*TP+FP*) (with FP meaning “false positive”) and *Recall* = *TP*/(*TP* + *FN*) (with FN meaning “false negative”). F1 scores range from 0 (worst) to 1 (best). The significance of classification scores was assessed using a permutation test (Ojala and Garriga, 2009).

## Results

### Behavioral Results

The median frequency with which videos were shared was 23% (range 3%–41%). After exclusion of outliers, the median response to decide whether or not to share a video was 0.98 ± 0.67 s (±SD, *n* = 2200). The response time was significantly shorter to decide to not share a video vs. to share a video (0.74 ± 0.944 s [*n* = 1655] vs. 0.95 ± 0.945 s [*n* = 545]; Mann-Whitney test, *U* = 344,878, *p* < 0.001, *r* = −0.18). We also found that S videos were significantly more liked than N-S videos (median score 0.75 vs. 0.45 on a 0–1 scale; Mann-Whitney test, *U* = 78,572, *p* < 0.001, *r* = −0.62).

### EEG Analysis: PSD and FAA

**Figure 2:**
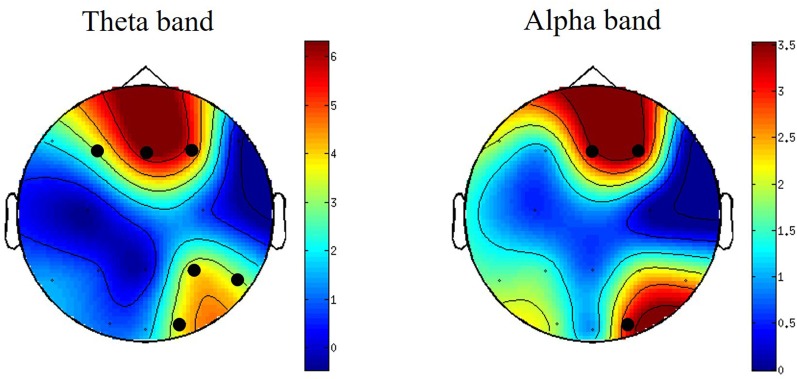
Topographically distributed independent-samples *t*-values for theta- and alpha-band power differences when watching S vs. N-S videos. For graphical purposes, the surface in between electrodes is interpolated to create scalp maps. Clusters with significant *t*-values are indicated by black circles. The colors bars indicate the *t*-values of the clusters. *N* = 23.

Differences between PSD oscillations (theta, alpha and beta) across frontal, central and parietal electrodes during viewing of S compared with N-S videos were assessed using non-parametric cluster-based permutation tests. No significant differences were detected for the time-frequency analysis. However, for channel-frequency (time averaged) analysis, significant differences were detected between S and N-S conditions. These differences were observed in midline-frontal and right parietal-occipital clusters for theta-band power (4–8 Hz) and in right-central frontal and right occipital clusters for alpha-band power (8–12 Hz; Figure 2). No significant differences were detected in beta-band clusters.

**Figure 3:**
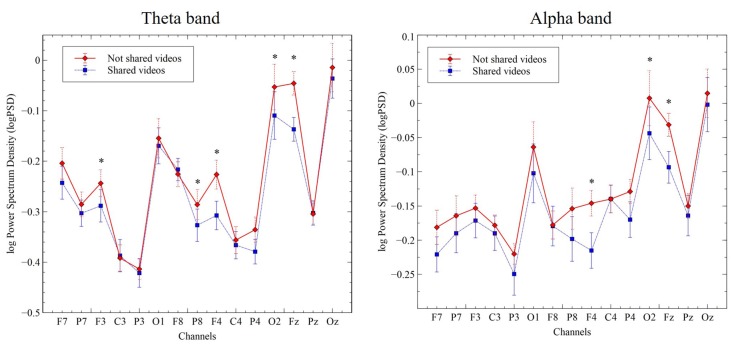
Change in log power spectral density (PSD; μV^2^/Hz) for the theta-band (4–8 Hz, left panel) and alpha-band (8–12 Hz, right panel) in the indicated channel clusters during viewing of S and N-S videos. Data represent the mean ± SEM. *p ≤ 0.05.

To examine the directionality of S vs. N-S-related changes in theta and alpha bands, we plotted the log-transformed PSD values for each electrode and computed their *t*- and *p-values* after correction for multiple comparisons (“Monte Carlo” method, 2000 randomizations). Theta-band, PSD values were significantly higher in the N-S condition, when compared with the S condition, for electrodes F3 (−0.24 vs. −0.29; *t* = 3.27, *p* = 0.018); Fz (−0.05 vs. −0.14; *t* = 6.34, *p* = 0.002); F4 (−0.22 vs. −0.31; *t* = 5.11, *p* = 0.002); P8 (−0.29 vs. −0.33; *t* = 3.16, *p* = 0.02); P4 (−0.34 vs. −0.38; *t* = 3.73, *p* = 0.004) and O2 (−0.05 vs. −0.11; *t* = 3.83, *p* = 0.004). Similarly, for alpha-band, PSD values were higher in the no-shared condition, when compared to the shared condition, for electrodes Fz (−0.03 vs. −0.09; *t* = 3.39, *p* = 0.010); F4 (−0.15 vs. −0.22; *t* = 3.53, *p* = 0.006) and O2 (0.01 vs. −0.04; *t* = 3.13, *p* = 0.022; Figure 3).

**Figure 4:**
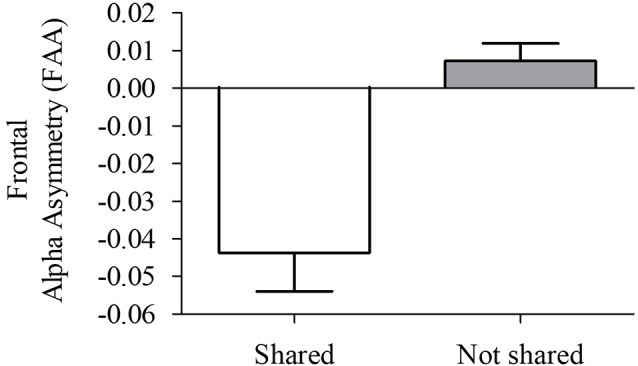
Frontal alpha asymmetry (FAA) index (lnPSD F4 − lnPSD F3) during viewing of S and N-S videos. Data are presented as the mean ± SEM (*t* = 4.95, *p* = 0.002). More negative FAA values reflect higher alpha activation in the right hemisphere when compared with the left hemisphere.

Since we observed significant differences in frontal alpha oscillations between the right and left hemispheres, the FAA index was computed. An independent-sample *t*-test showed a significant difference between S and N-S conditions (*t* = −4.95, *p* = 0.002, “Monte Carlo” method, 2000 times randomizations). A more negative FAA score obtained for viewing S videos compared with N-S videos (Figure 4), reflecting higher right frontal alpha activity.

### Classification Results

To determine whether the neural data obtained with our subjects could improve prediction of sharing intention, we compared classification models using SVM. Using only the pleasantness rating (self-reported variable) as a feature, our classifier accuracy was 78% and the F1 Score was 0.78 (F1 score reaches its best value at 1 and worst at 0, *p* < 0.01, permutation test, 100 iterations, Table 1; F1 scores of 1 and 0 reflect the best and worst scores, respectively). However, combining the self-reported ratings with the theta- and alpha-band PSDs and the FAA index as features increased the classifier accuracy to 81% and the F1 Score of 0.81 (*p* < 0.01, permutation test, 100 iterations), representing a significant improvement in the general accuracy of 3% (*t* = 2.75, *p* = 0.005, *t-test* between the predictions made by the two models using the test data, Table 2). These results suggest that neural markers may be useful parameters in evaluating sharing intention.

**Table 1:** Classification results using only pleasantness ratings (self-reported variable).

Condition	Precision	Recall (Accuracy)	F1 score
Share	0.78	0.80	0.79
Not shared	0.79	0.77	0.78
Total/Avg	0.78	0.78	0.78

**Table 2:** Classification results using pleasantness ratings (self-reported variable) plus EEG parameters.

Condition	Precision	Recall (Accuracy)	F1 score
Share	0.78	0.86	0.82
Not shared	0.85	0.75	0.80
Total/Avg	0.81	0.81	0.81

## Discussion

In the present study, we investigated correlations between EEG-derived data and information sharing behavior, using viewing of videos that would or would not be shared as the model system. Time-frequency analysis was conducted revealing no significant differences between conditions (S vs. N-S videos). EEG channel-frequency analysis revealed that viewing of N-S videos resulted in higher PSD amplitudes in the theta band, primarily over bilateral frontal (F3, F4 and Fz), right parietal (P4 and P8) and right occipital (O2) sites and higher PSD amplitudes in the alpha band over central-right frontal sites (F4 and Fz) and right occipital (O2) sites compared with viewing of S videos. These data are consistent with the lower FAA index (i.e., higher right frontal brain activity) for viewing of S vs. NS videos. We also discovered that these EEG measures could aid in the prediction of sharing behavior. Using SVM, a machine-learning algorithm widely used in neuroscience applications (Quitadamo et al., 2017), we found that a self-reported variable in combination with theta PSD, alpha PSD and FAA index as features improved the prediction accuracy by 3% compared with the self-reported variable alone. Therefore, these data have identified an EEG oscillation pattern that can improve the prediction of information sharing behavior.

First we found that theta rhythms in bilateral frontal and right parietal and occipital sites were higher in N-S trials, when compared to the S trials. Previous studies have yielded conflicting evidence for the contribution of theta-band power to cognitive processes. Klimesch (1999) proposed that theta-band power could be negatively related to cognitive and memory performance and brain maturity, but in a complex and partly non-linear manner. The author states that within the theta frequency range, EEG power is negatively related to cognitive performance, whereas the theta reactivity (or more specifically theta synchronization) is related to a good cognitive performance and memory. Additional studies have shown that theta frequency activity is increased, especially at frontal sites, during cognitive activities that require attention or short-term memory, such as mental arithmetic and working memory load tasks, which indicates a possible role of theta oscillations in working memory maintenance (reviewed by Scheeringa et al., 2008). This also hints at the possibility that information sharing behavior might be associated with higher theta amplitudes, particularly at frontal sites, since this behavior demands engagement of higher cognitive processes. Falk and Scholz (2018) noted that the decision to share information would require increased externalized attention, given that this behavior involves integration of brain processes related to stimuli value computation, self-related relevance and inference of another’s state-of-mind. However, other studies have shown that more generalized theta rhythm activation pattern (i.e., not restricted at frontal sites) can be related to more automatic processes and diminished attentional efforts. Braboszcz and Delorme (2011) showed that among participants performing a breath-counting task, generalized theta EEG activity, especially within occipital and parieto-central regions, was increased during mind-wandering moments. Thus, higher theta activity could reflect decreased alertness and sensory processing when participants are disengaged from the task at hand. Hermens et al. (2005) demonstrated that patients with attention deficit hyperactivity disorder displayed higher theta power that correlated with poorer performance in an attentional (oddball) task compared with control subjects. Lin et al. (2016) also demonstrated a relationship between higher theta power and declining attentional effort and worsening of task performance. Based on these observations, we suggest that in our study, the higher theta PSD pattern along right frontal, parietal and occipital sites and the lower response times associated with the decision to not share a video reflect a lower cognitive demand, thereby generating a quicker and more automatic decision.

We also found that right frontal alpha rhythms are associated with information sharing intention. EEG alpha rhythms, which were first identified by Berger (1929), have been interpreted by some authors to be an “idling” rhythm with a diminished amplitude when the eyes are opened or mental activity is engaged (Laufs et al., 2003). Consequently, high and low alpha power is typically associated with low and high mental activity, respectively (Goldman et al., 2002). It has been shown that frontal asymmetries have physiological relevance for inferring emotional behavioral patterns (for an extensive review, see Harmon-Jones et al., 2010; Smith et al., 2017) and are involved in motivation driven-behaviors. Given this, our finding of alpha power asymmetry prompted us to compare the FAA indexes obtained under S vs. N-S video viewing conditions. We found that FAA scores were more negative (higher right frontal brain activity) when viewing S compared with N-S videos, which was somewhat surprising because one might expect information sharing behavior to be more associated with approach-related behavior and thus with higher frontal left-brain activity (Coan and Allen, 2003). Various studies have shown that information sharing decisions and successful persuasive or influential behavior involve increased activity in “valuation network”-related brain areas, such as the ventral striatum and ventromedial prefrontal cortex (vmPFC; Falk et al., 2013; Baek et al., 2017; Scholz et al., 2017). In addition, it has been proposed that there may be some intrinsic reward or positive value in information sharing (Tamir and Mitchell, 2012; Falk et al., 2013). However, it is important to note that the value assigned to a particular choice or action is subjective and involves a range of features dependent on the individual and social context. The brain’s value system may thus be involved in tracking non-compliance vs. conformity with group opinion rather than simply the stimulus value. Indeed, some evidence suggests that the brain initially computes convergence and divergence with group opinion as the main goal (Falk and Scholz, 2018). Schnuerch and Pfattheicher (2017) showed that susceptibility to social influence (e.g., social conformity) is mainly guided by higher punishment sensitivity and chronic avoidance behavior. Thus, the pressure to conform is related to avoidance of detrimental consequences of disagreement. These authors also showed that the tendency to adopt the group’s response was negatively associated with FAA, indicating that stronger right hemisphere activation is associated with higher social conformity. This finding supports the notion that social conformity is guided by avoidance-related rather than approach-seeking behavior. Our finding that higher right frontal brain activity is related to information sharing behavior is therefore consistent with the hypothesis that information sharing is driven by social conformity and avoidance of negative outcomes of non-compliance with a larger group, such as social isolation. However, further research is necessary to assess the contribution of reward vs. punishment processes to information sharing behavior.

A third finding of our study is that EEG data features combined with a subjective variable (like/dislike score) improved the accuracy of information sharing intention by 3% compared with the subjective variable alone. Other authors have examined the predictive value of neuronal activity parameters using videos as the stimuli. For example, Dmochowski et al. (2014) examined EEG parameters for subjects who viewed a recorded television broadcast and used inter-subject correlation analysis to analyze the association between EEG parameters and social media metrics (viewership size and tweet frequency rates). They found that the social media interactions during commercial breaks could predict the preference rating for each advertisement after broadcasting. Similarly, Boksem and Smidts (2015) compared the ability of neural measures and traditional self-stated preference measures to predict the box-office success of movies. Their results indicated that overall beta and gamma activity in EEGs provided predictive information about individual preferences and population-wide success, respectively. Although the predictive power of the neuronal measures was small (explained variance <2%), the magnitude is comparable to the 3% improvement we obtained here. Interestingly, several recent studies have discussed the use of neurophysiological measures to improve brain state classification and its potential applications in brain-machine interfaces and prediction of behavioral outcomes (Hettich et al., 2016; Gauba et al., 2017; So et al., 2017; Zhao et al., 2018). Our work therefore provides additional support for the utility of EEG features in improving prediction of information sharing intention.

Our study has several limitations. First, we couldn’t detect significant differences regarding time-frequency analysis between S vs. N-S videos. We suspect that this negative result happened because we conducted our analysis concerning the answers that were given to each trial (video). It means the trials within each condition were heterogeneous regarding the videos they contained. It could lead to more complex frequency oscillations patterns through time which could reduce the chances to observe subtle effects between the two conditions (S vs. N-S). We also suspect that movement-related artifacts due to the long experimental sessions could have contributed to blur possible time-frequency effects that might have arisen. Second, recording of EEGs for sustained periods (>40 min in our study) when head movements are not restricted can increase the number of artifacts. This effect is exacerbated by low electrode density recordings (<64 electrodes) because it limits the number of artifacts that can be discarded after independent component analysis (ICA) and increases the removal of noisier epochs and even whole channels. Here, we used 21 channels and rejected 37.7% of trials, which is comparable to the 25% trial rejection rate of Braboszcz and Delorme (2011), using 128 channels and long (20 s) trials of a simple breath-counting task. Nevertheless, the 3% predictive improvement in our study, which could potentially be improved using high-density recordings, demonstrates the power of EEG measures as potential candidates to explain sharing behavior outcomes. Third, this was an exploratory study without *a priori* assumption evaluate traditional neural markers. Our only goal was to determine whether the three frequency bands (theta, alpha and beta) might be involved in information sharing decisions, and we had no preconceived expectations of what we might observe. This approach can increase the number of statistical comparisons leading to type I error. However, we addressed this issue by implementing nonparametric permutation testing as described by Maris and Oostenveld (2007), which accounts for issues related to the multiple comparison problem, especially in exploratory studies.

Despite its limitations, the present study makes several valuable contributions to the understanding of social neuroscience dynamics. As stated earlier, the sense of belonging to a society and social coordination is critical for human well-being and survival (Baumeister and Leary, 1995; Lieberman and Eisenberger, 2009). Influencing opinions and ideas is a core mechanism by which different groups interact and communicate. Consequently, diverse social, emotional and cognitive processes must be integrated, often without conscious awareness, when sharing information. Combining neural activity and behavioral measures with computational approaches can help to integrate and understand the underlying mechanisms that drive information sharing behavior, and thus improve the predictive ability of statistical models of sharing. This powerful knowledge has applications in domains such as marketing research (Berns et al., 2010; Ohme et al., 2010; Pozharliev et al., 2017), economic decisions (Camerer, 2008; Weber and Johnson, 2009) and social media and public health campaigns (Falk et al., 2010, 2012, 2015; Cascio et al., 2013). In conclusion, our identification of neural markers related to sharing information behavior may improve our understanding of the underlying motives and mechanisms of such behavior. We also demonstrate that analysis of neural oscillations in message propagation research can improve the predictive strength of self-reported measures.

## Author Contributions

NF and MF designed the study. NF performed the experiments and interpreted the results. NF and RP pre-processed the data and wrote the article. NF, RP and MF analyzed the data. MF reviewed and contributed to the final version. All authors read and approved the submitted version.

## Conflict of Interest Statement

The authors declare that the research was conducted in the absence of any commercial or financial relationships that could be construed as a potential conflict of interest.

## Footnotes

^1^http://mashable.com/2013/12/27/viral-videos-2013/#DFE9zvjpOkqw
